# Supplementary material for: Chemical Constituents of the Leaves of Peltophorum pterocarpum and Their Bioactivity
Source: Molecules. 2019 Jan 10;24(2):240. doi: 10.3390/molecules24020240 (PMC6359222; doi:10.3390/molecules24020240)

Supplementary Materials

Chemical Constituents from the Leaves of *Peltophorum pterocarpum* and Their Bioactivity

Yue-Chiun Li [1], Ping-Chung Kuo [2,]*, Mei-Lin Yang [3], Tzu-Yu Chen [3], Tsong-Long Hwang [4], Chih-Chao Chiang [5], Tran-Dinh Thang [6], Nguyen Ngoc Tuan [7], and Jason T.C. Tzen [1,]*

[1] Graduate Institute of Biotechnology, National Chung-Hsing University, Taichung 402, Taiwan; ycli0126@gmail.com (Y.-C.L.)

[2] School of Pharmacy, College of Medicine, National Cheng Kung University, Tainan 701, Taiwan

[3] Department of Biotechnology, National Formosa University, Yunlin 632, Taiwan; black4635@gmail.com (T.-Y.C.), L3891104@nckualumni.org.tw (M.-L.Y.)

[4] Graduate Institute of Natural Products, College of Medicine, Chang Gung University; Research Center for Industry of Human Ecology, Research Center for Chinese Herbal Medicine, and Graduate Institute of Health Industry Technology, Chang Gung University of Science and Technology; Department of Anesthesiology, Chang Gung Memorial Hospital, Taoyuan 333, Taiwan; htl@mail.cgu.edu.tw

[5] Graduate Institute of Clinical Medical Sciences, College of Medicine, Chang Gung University; Supervisor board, Taoyuan Chinese Medicine Association; Dazhu Fengze Chinese Medicine Clinic, Taoyuan 338, Taiwan; moonlight0604@hotmail.com

[6] School of Chemistry, Biology and Environment, Vinh University, Vinh City, Vietnam; thangtd@vinhuni.edu.vn

[7] Institute of Biotechnology and Food Technology, Industrial University of Ho Chi Minh City, Ho Chi Minh City, Vietnam; nguyenngoctuan@iuh.edu.vn

* Correspondence: z10502016@email.ncku.edu.tw (P.-C.K.), tctzen@dragon.nchu.edu.tw (J.T.-C.T.); Tel.: +886-6-2353535 # 6806 (P.-C.K.), +886-4-22840328 (J.T.-C.T.).

Contents

S1. Extraction and isolation schemes

S2. Anti-inflammatory bioactivity experimental procedures

Fig. S1. ^{1}H NMR spectrum of **1**

Fig. S2. ^{13}C and DEPT NMR spectrum of **1**

Fig. S3. HMBC spectrum of **1**

Fig. S4. NOESY spectrum of **1**

Fig. S5. Expanded NOESY spectrum of **1**

Fig. S6. MS/HRMS spectra of **1**

Fig. S7. ^{1}H NMR spectrum of **2**

Fig. S8. ^{13}C and DEPT NMR spectrum of **2**

Fig. S9. HMBC spectrum of **2**

Fig. S10. NOESY spectrum of **2**

Fig. S11. Expanded NOESY spectrum of **2**

Fig. S12. HRMS spectrum of **2**

Fig. S13. ^{1}H NMR spectrum of **2** (recorded in C_6D_6)

Fig. S14. Expanded NOESY spectrum of **2** (recorded in C_6D_6)

Fig. S15. ^{1}H NMR spectrum of **51**

Fig. S16. ^{13}C and DEPT NMR spectrum of **51**

Fig. S17. ^{1}H NMR spectrum of **52**

Fig. S18. ^{13}C and DEPT NMR spectrum of **52**

S1. Extraction and isolation schemes

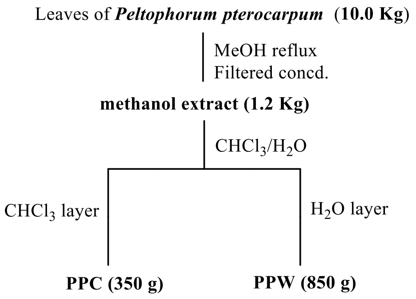

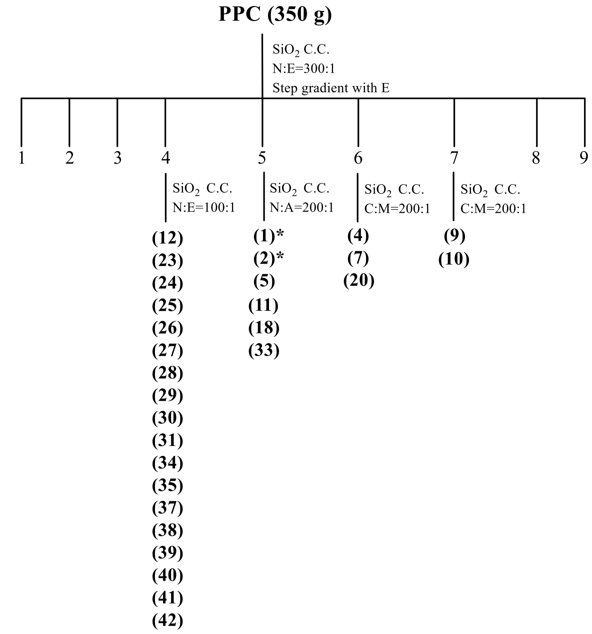

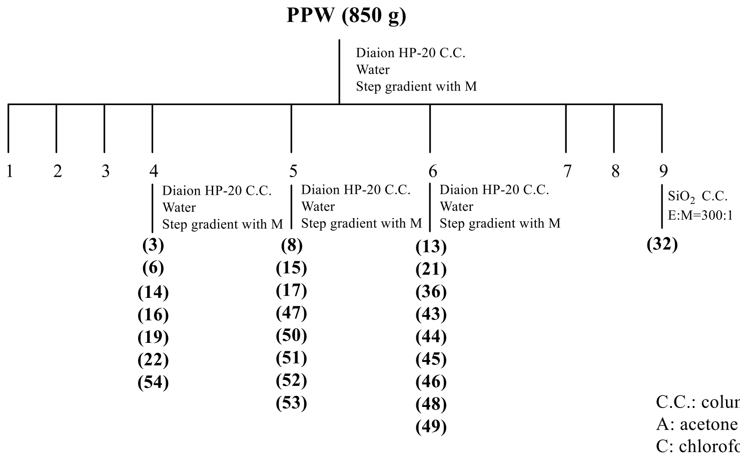

C.C.: column chromatography
A: acetone
C: chloroform
E: ethyl acetate
N: *n*-hexane
M: methanol
*: new compound

S2. Anti-inflammatory bioactivity experimental procedures

Preparation of Human Neutrophils. A study involving human neutrophils was approved by the Institutional Review Board at Chang Gung Memorial Hospital, Taoyuan, Taiwan, and was conducted according to the Declaration of Helsinki (2013). The written informed consent was obtained from each healthy donor before blood was drawn. Blood was drawn from healthy human donors (20–30 years old) by venipuncture into heparin-coated vacutainer tubes, using a protocol approved by the Institutional Review Board at Chang Gung Memorial Hospital. Blood samples were mixed gently with an equal volume of 3 % dextran solution. Neutrophils were isolated with a standard method of dextran sedimentation prior to centrifugation in a Ficoll Hypaque gradient and hypotonic lysis of erythrocytes. The leukocyte-rich plasma was collected after sedimentation of the red cells for 30 min at room temperature, and was transferred to 20 mL Ficoll solution (1.077 g/mL) and spun down at 400 g for 40 min at 20 °C. The granulocyte/ erythrocyte pellets were resuspended in ice-cold 0.2 % NaCl to lyse erythrocytes. After 30 sec, the same volume of 1.6 % NaCl solution was added to reconstitute the isotonic condition. Purified neutrophils were pelleted and then resuspended in a calcium (Ca^{2+})-free Hank's balanced salt solution (HBSS) buffer at pH 7.4, and were maintained at 4 °C before use.

Measurement of Superoxide Anion Generation. The assay of the generation of superoxide anion was based on the SOD-inhibitable reduction of ferricytochrome c. In brief, after supplementation with 0.5 mg/mL ferricytochrome c and 1 mM Ca^{2+}, neutrophils (6×10^5 cells/mL) were equilibrated at 37 °C for 2 min and incubated with drugs or an equal volume of vehicle (0.1 % DMSO, negative control) for 5 min. Cells were activated with 100 nM fMLP during the preincubation of 1 μg/mL cytochalasin B (fMLP/CB) for 3 min. Changes in the absorbance with a reduction in ferricytochrome c at 550 nm were continuously monitored

in a double-beam, six-cell positioner spectrophotometer with constant stirring (Hitachi U-3010, Tokyo, Japan). Calculations were based on differences in the reactions with and without SOD (100 U/mL) divided by the extinction coefficient for the reduction of ferricytochrome c (ε = 21.1/mM/10 mm).

Measurement of Elastase Release. Degranulation of azurophilic granules was determined by elastase release as described previously. Experiments were performed using MeO-Suc-Ala-Ala-Pro-Val-*p*-nitroanilide as the elastase substrate. Briefly, after supplementation with MeO-Suc-Ala-Ala-Pro-Val-*p*-nitroanilide (100 μM), neutrophils (6×10^5/mL) were equilibrated at 37 °C for 2 min and incubated with test compounds or an equal volume of vehicle (0.1 % DMSO, negative control) for 5 min. Cells were activated by 100 nM fMLP and 0.5 μg/mL cytochalasin B, and changes in absorbance at 405 nm were continuously monitored to assay elastase release. The results were expressed as the percent of elastase release in the fMLP/CB-activated, drug-free control system.

Statistical Analysis. All the experiments were performed in triplicate (n=3). Results were expressed as mean ± S.E.M. Statistical comparisons were made between groups using the Student's *t* test. Values of *p* less than 0.05 were considered to be statistically significant, and * $p < 0.05$, ** $p < 0.01$, *** $p < 0.001$, respectively.

Fig. S1. ^{1}H NMR spectrum of **1**

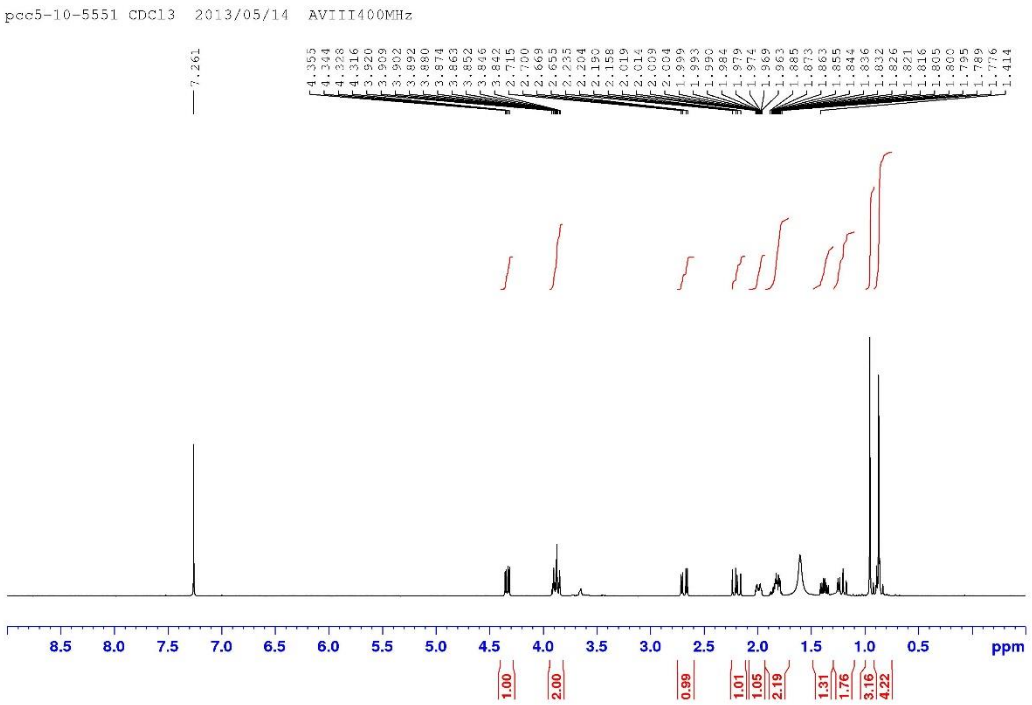

Fig. S2. ^{13}C and DEPT NMR spectrum of **1**

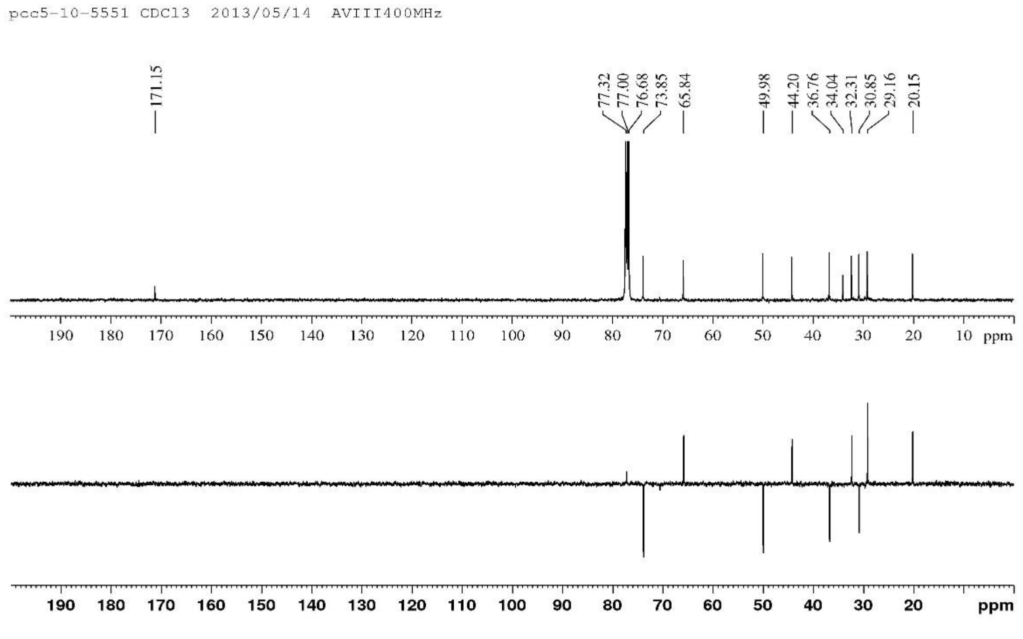

Fig. S3. HMBC spectrum of **1**

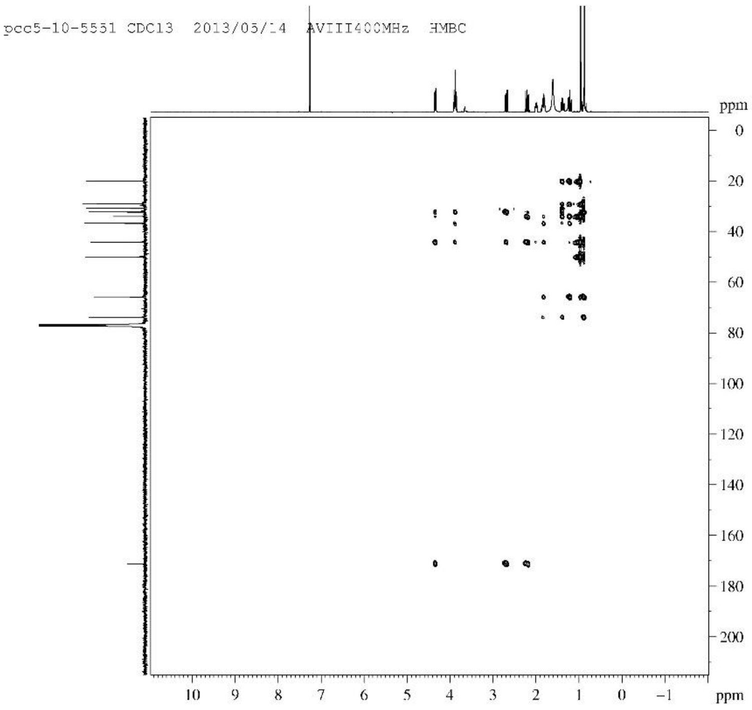

Fig. S4. NOESY spectrum of **1**

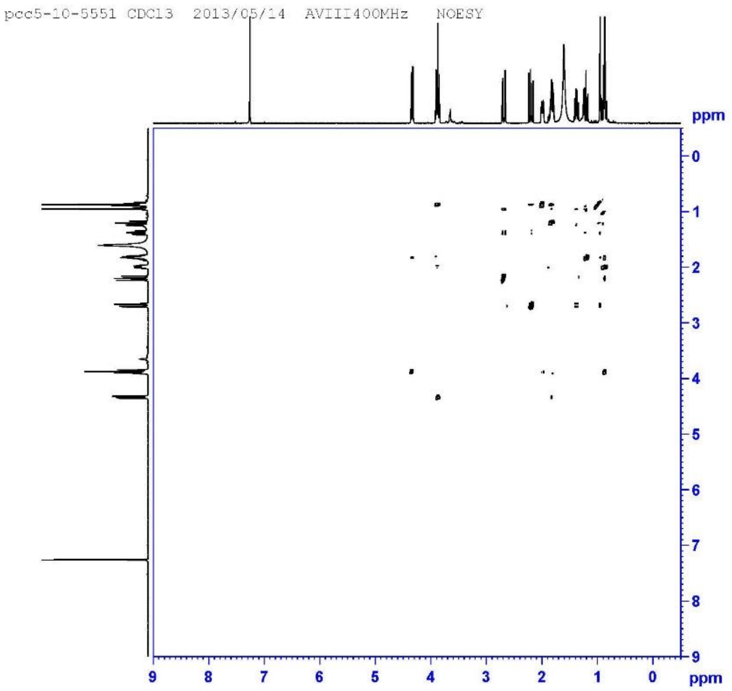

Fig. S5. Expanded NOESY spectrum of **1**

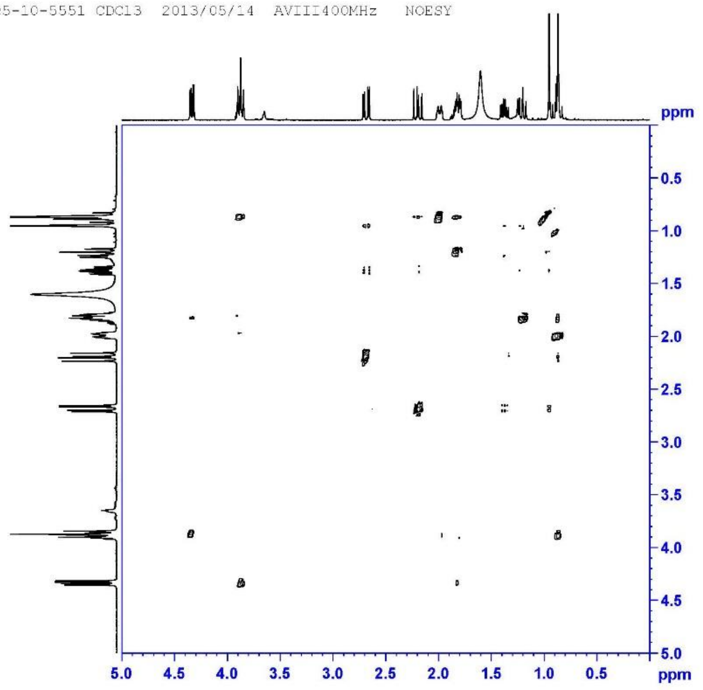

Fig. S6. MS/HRMS spectra of **1**

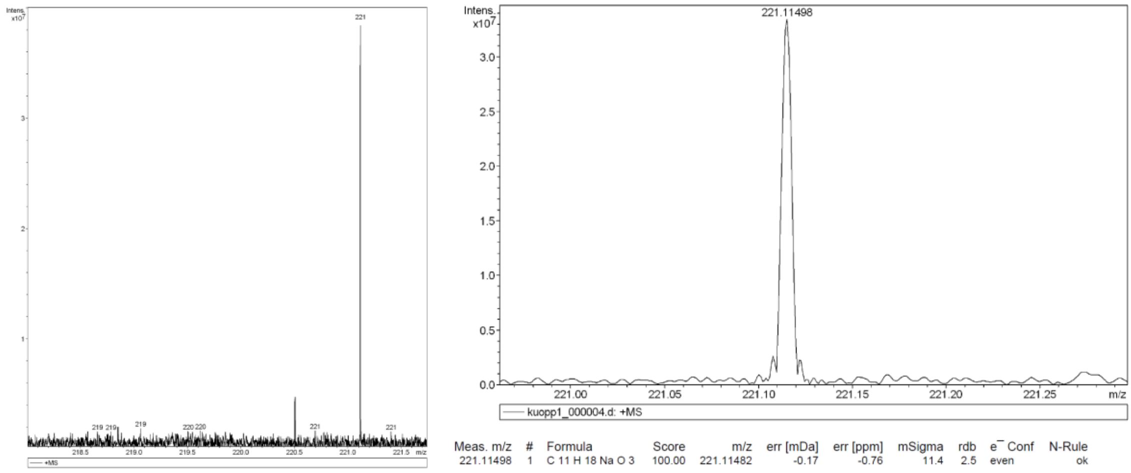

Meas. m/z	#	Formula	Score	m/z	err [mDa]	err [ppm]	mSigma	rdb	e⁻ Conf	N-Rule
221.11498	1	C 11 H 18 Na O 3	100.00	221.11482	-0.17	-0.76	11.4	2.5	even	ok

Fig. S7. ^{1}H NMR spectrum of **2**

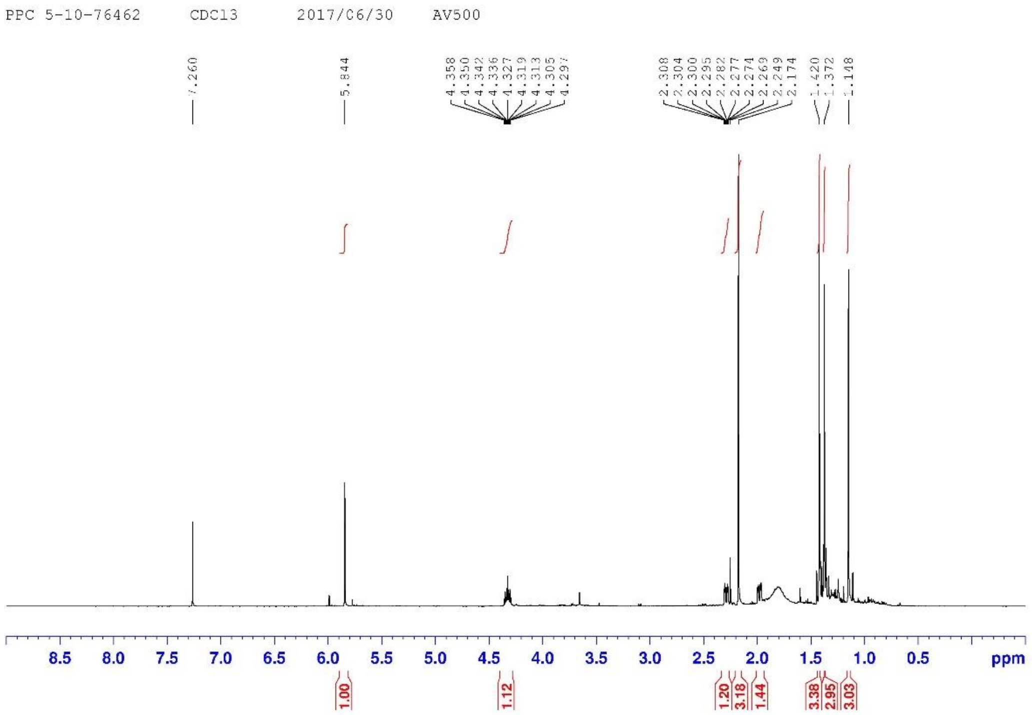

Fig. S8. ^{13}C and DEPT NMR spectrum of **2**

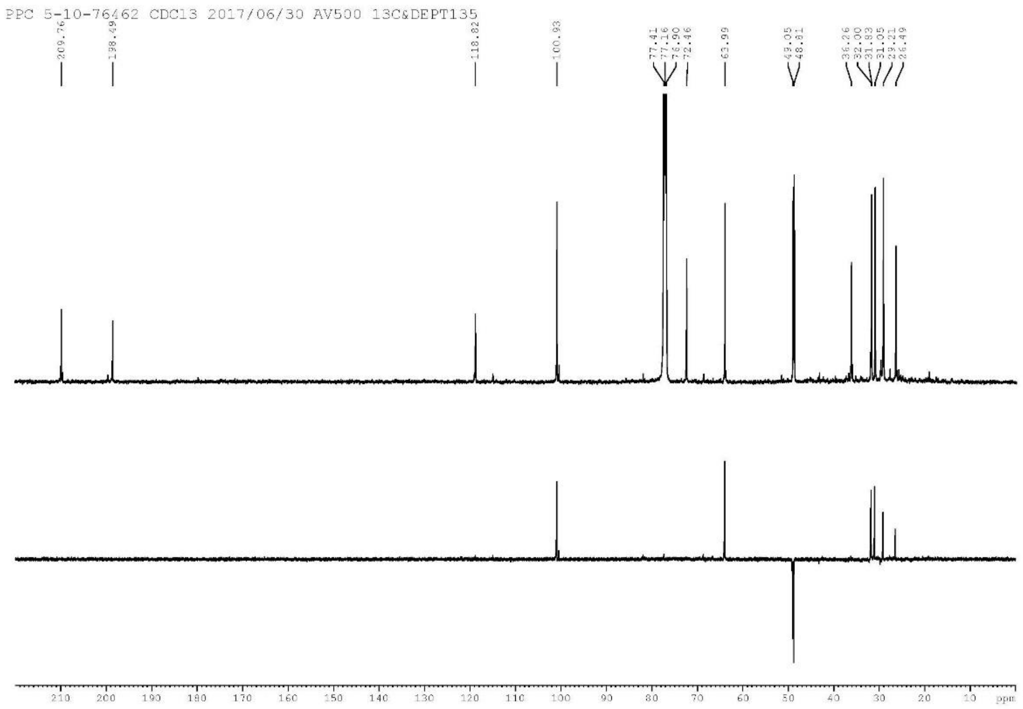

Fig. S9. HMBC spectrum of **2**

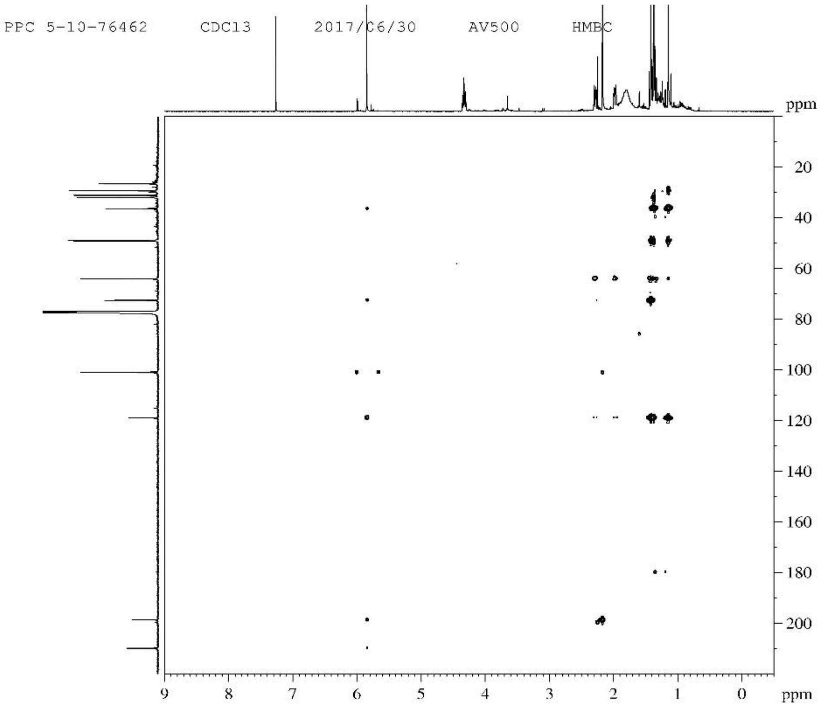

Fig. S10. NOESY spectrum of **2**

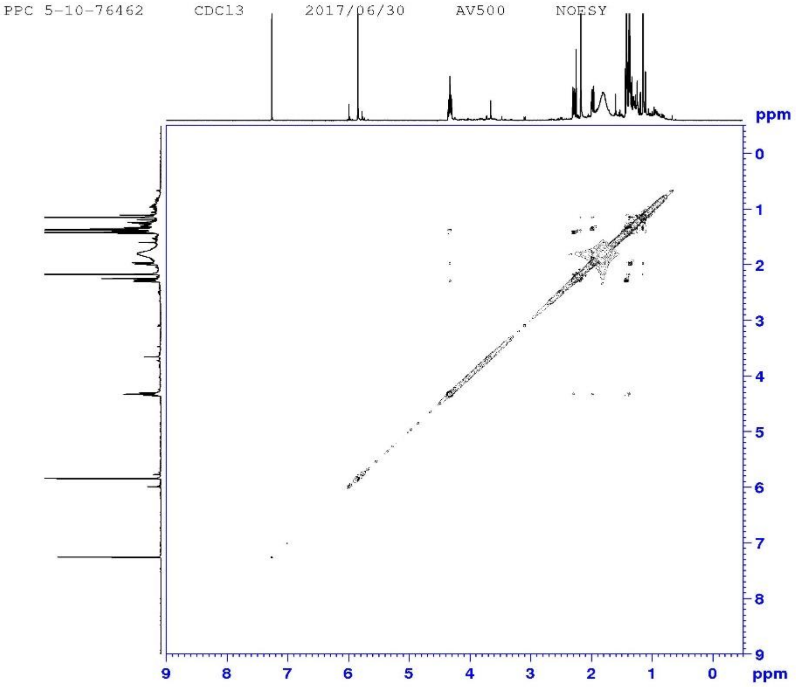

Fig. S11. Expanded NOESY spectrum of **2**

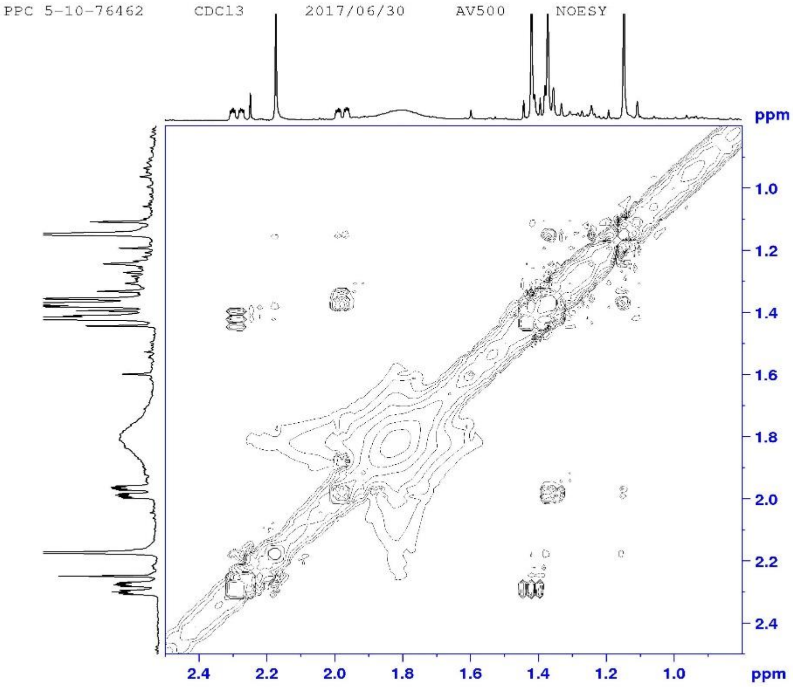

Fig. S12. HRMS spectrum of **2**

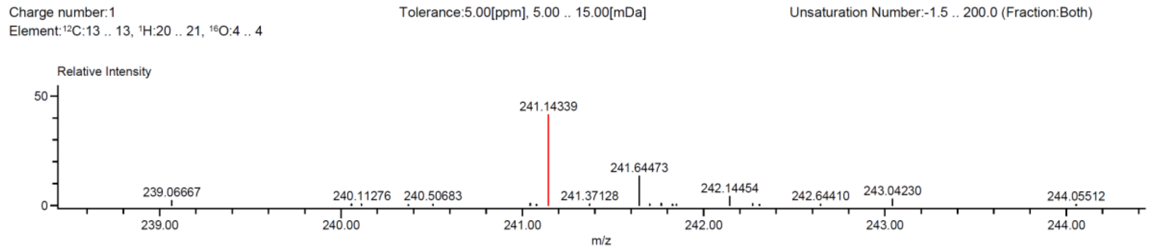

Mass	Intensity	Calc. Mass	Mass Difference [mDa]	Mass Difference [ppm]	Possible Formula	Unsaturation Number
241.14339	46424.25	241.14398	-0.59	-2.46	$^{12}C_{13}{}^{1}H_{21}{}^{16}O_4$	3.5

Fig. S13. ^{1}H NMR spectrum of **2** (recorded in C_6D_6)

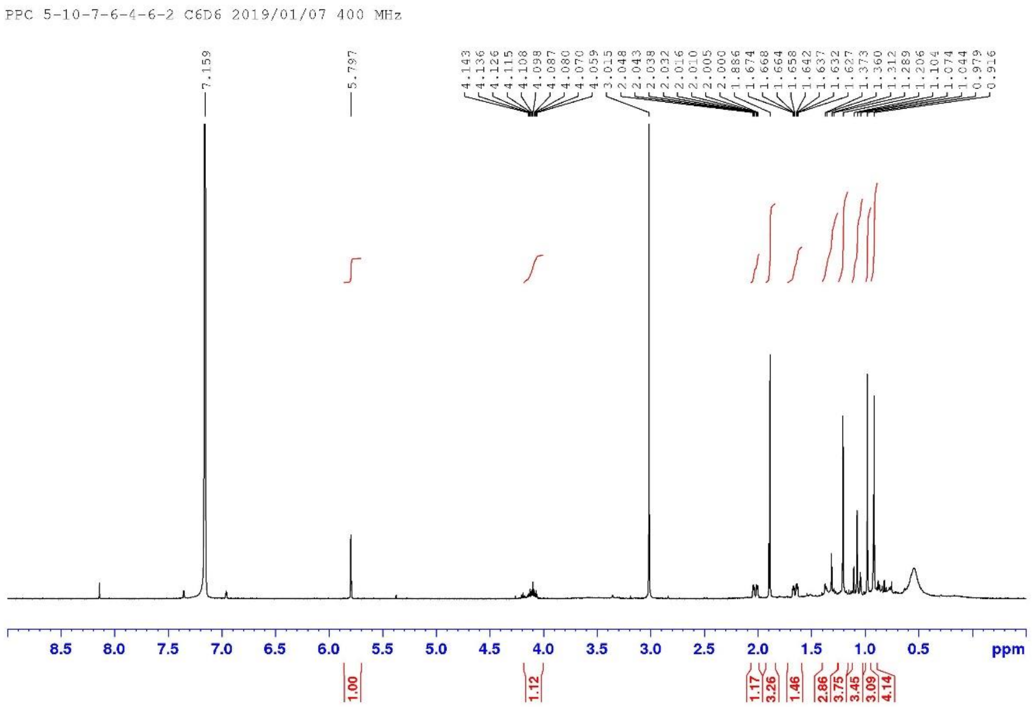

Fig. S14. Expanded NOESY spectrum of **2** (recorded in C_6D_6)

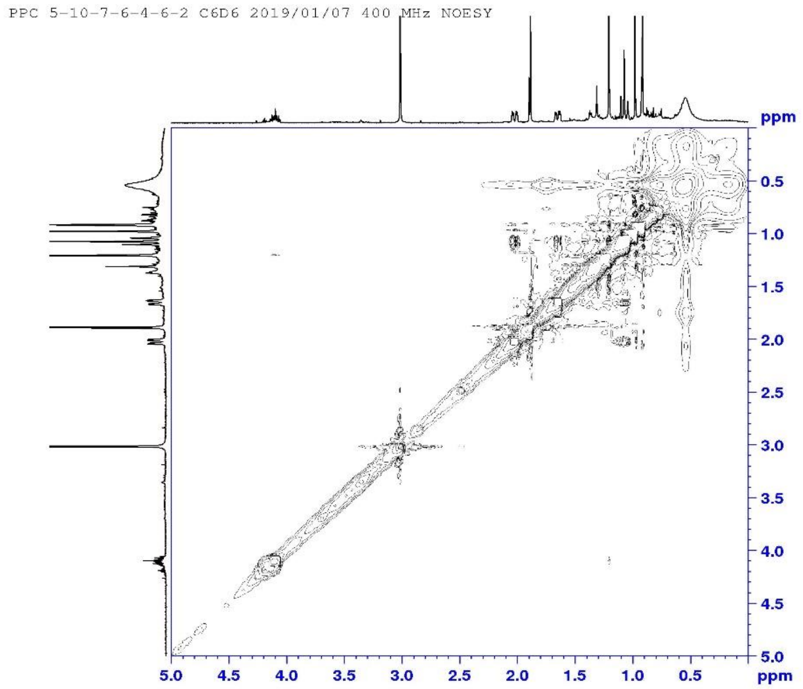

Fig. S15. ^{1}H NMR spectrum of **51**

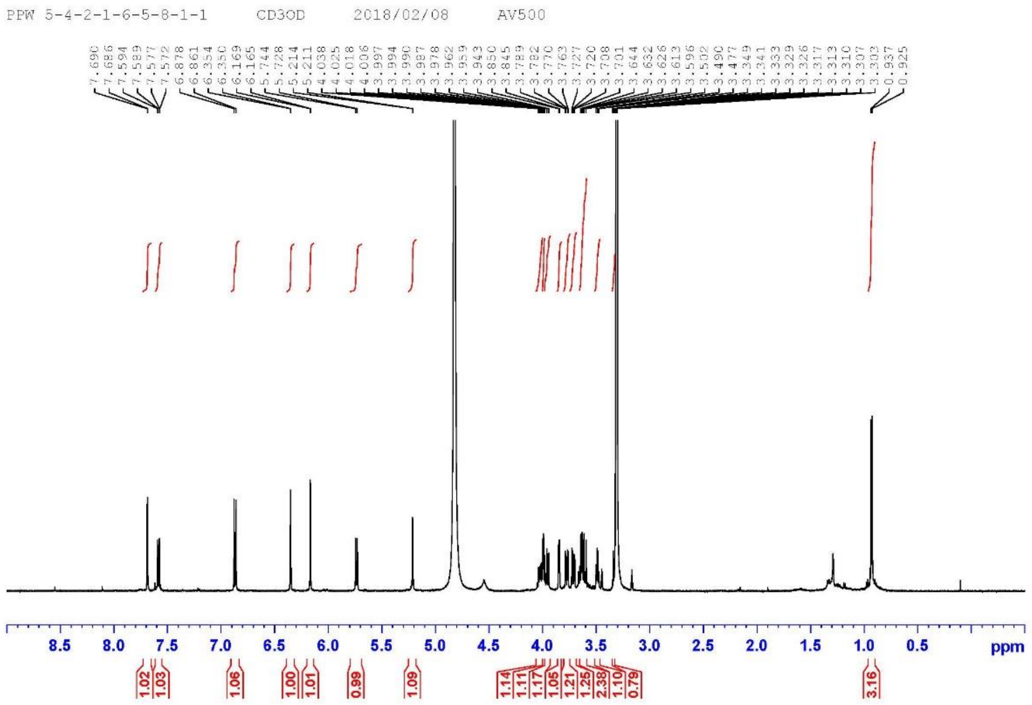

Fig. S16. ^{13}C and DEPT NMR spectrum of **51**

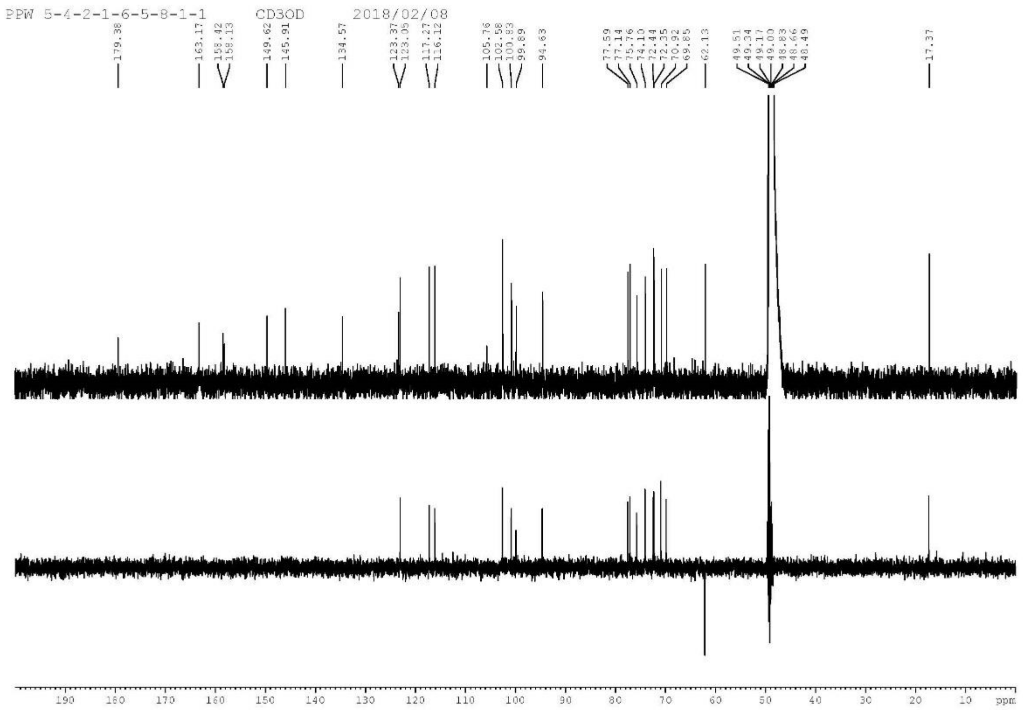

Fig. S17. ^{1}H NMR spectrum of **52**

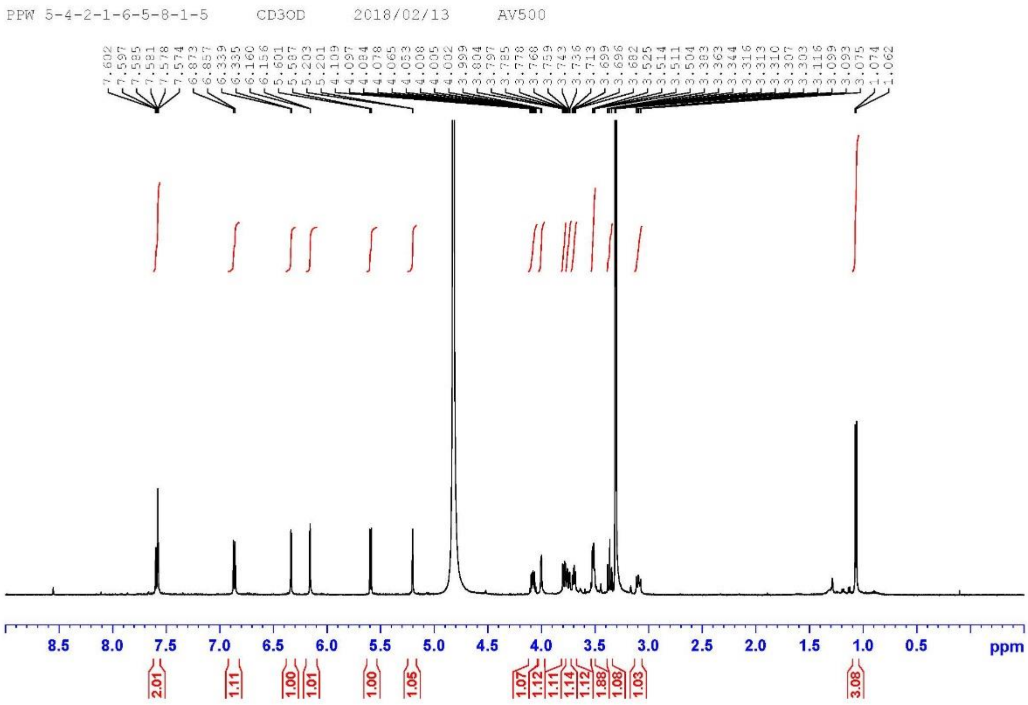

Fig. S18. ^{13}C and DEPT NMR spectrum of **52**

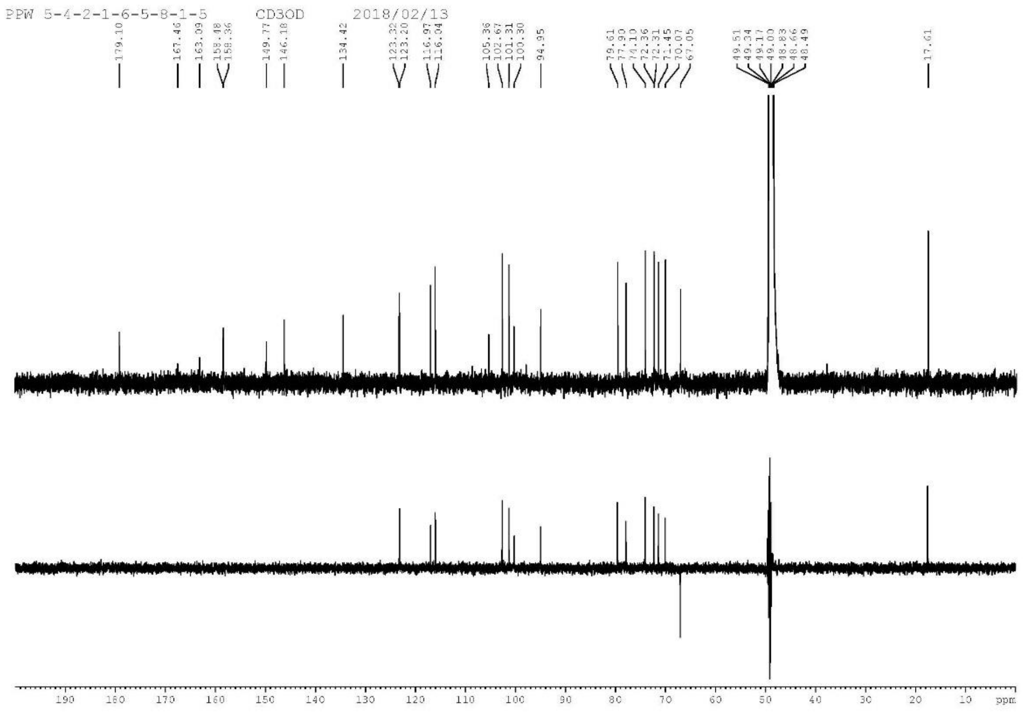

Supplement: Supplementary file 1 [file molecules-24-00240-s001.pdf]
